# A noval prognostic signature of the N7-methylguanosine (m7G)-related miRNA in lung adenocarcinoma

**DOI:** 10.1186/s12890-022-02290-7

**Published:** 2023-01-12

**Authors:** Han-ping Duan, Jian-hui Yan, Lin Nie, Ye Wang, Hui Xie

**Affiliations:** 1 Department of Nuclear Medicine, Affiliated Hospital (Clinical College) of Xiangnan University, Chenzhou, 423000 Hunan Province People’s Republic of China; 2 Department of General Medicine, Affiliated Hospital (Clinical College) of Xiangnan University, Chenzhou, 423000 Hunan Province People’s Republic of China; 3 Department of Radiology, Affiliated Hospital (Clinical College) of Xiangnan University, Chenzhou, 423000 Hunan Province People’s Republic of China; 4 Department of Thoracic Surgery, Affiliated Hospital (Clinical College) of Xiangnan University, Chenzhou, 423000 Hunan Province People’s Republic of China; 5 Department of Radiation Oncology, Affiliated Hospital (Clinical College) of Xiangnan University, No. 25, Renmin West Road, Chenzhou, 423000 Hunan Province People’s Republic of China; 6 Key Laboratory of Medical Imaging and Artifical Intelligence of Hunan Province, 423000 Chenzhou, People’s Republic of China

**Keywords:** Lung adenocarcinoma, N7-methylguanosine, miRNA

## Abstract

**Background:**

Lung adenocarcinoma (LUAD) is characterized by high morbidity and mortality rates and poor prognosis. N7-methylguanosine play an increasingly vital role in lung adenocarcinoma. However, the prognostic value of N7-methylguanosine related-miRNAs in lung adenocarcinoma remains unclear.

**Methods:**

In the study, the mRNA and miRNA expression profiles and corresponding clinical informations were downloaded from the public database. The prognostic signature was built using least absolute shrinkage and selection operator Cox analysis. The Kaplan–Meier method was used to compare survival outcomes between the high- and low-risk groups. Signatures for the development of lung adenocarcinoma were tested using univariate and multivariate Cox regression models. Single-sample gene set enrichment analysis was used to determine the immune cell infiltration score. First, we predicted METTL1 and WDR4 chemosensitivities based on a public pharmacogenomics database. The area under the receiver operating characteristic curve showed that the performance of signature in 1-,3-, and 5-year survival predictions were 0.68, 0.65, and 0.683, respectively.

**Results:**

We established a novel prognostic signature consisting of 9 N7-Methylguanosine related miRNAs using least absolute shrinkage and selection operator Cox analysis. Patients in the high-risk group had shorter survival times than those in the low-risk group did. The calibration curves at 1, 3, and 5-year also illustrate the high predictive power of the structure. Signature was corrected using the Toumor stage. The expression levels of METTL1 and WDR4 significantly correlated with the sensitivity of cancer cells to antitumor drugs.

**Conclusions:**

A novel signature constructed using 9 N7-methylguanosine related-miRNAs can be used for prognostic prediction.

**Supplementary Information:**

The online version contains supplementary material available at 10.1186/s12890-022-02290-7.

## Introduction

Human health is primarily threatened by lung adenocarcinoma (LUAD), which causes high morbidity and mortality [1]. Lung cancer is the second most common and fatal cancer worldwide, according to data released in 2021 [2]. According to statistics, the main trend in the incidence of lung cancer in recent years is that lung adenocarcinoma (LUAD) has increased and lung squamous carcinoma (LUSA) has decreased annually [2]. At the present time, the mechanisms of lung cancer occurrence and development are not entirely understood. Thus, further understanding of the mechanisms that contribute to the development and prevention of lung cancer is urgently required.

N7-methylguanosine (m7G) is the most ubiquitous RNA cap modification [3] and also functions in transcription, mRNA splicing, and translation [4, 5]. Recent studies have shown that m7G plays an increasingly vital role in human diseases, especially cancer [6]. It has recently been found that methyltransferase-like 1(METTL1)-mediated m7G methylation affects the stability of the stem-loop structure by disrupting the RNA G-quadruplexs (G4s), thereby promoting its process from pri- to pre-miRNA, ultimately leading to miRNA maturation [7]. Mature miRNAs can function as oncogenes or tumor suppressors by binding to downstream target genes [8]. Moreover, whether these m7G-related miRNAs affect lung cancer prognosis remains unknown.

In the present study, we identified m7G-related mRNAs. We used the public prediction platform TargetScan (TargetScan Release 7.2, URL: http://www.targetscan.org/) to search for m7G-related miRNAs. M7G-related miRNAs were systematically analyzed using bioinformatic methods, and important miRNAs were selected to construct a prognostic model for LUAD.

## Materials and methods

### Data collection

Raw data were downloaded from a public database website (https://portal.gdc.cancer.gov/projects/tcga). It contains mRNA sequencing information of 497 cases of LUAD and 54 cases of normal lung tissue, miRNA sequencing information of 521 cases of LUAD and 46 cases of normal lung tissue, as well as the corresponding clinical data. Two m7G-related mRNAs, METTL1 and WD-repeat domain 4 (WDR4), were obtained from related studies [9].

### Construction and validation of a M7G-related miRNA signature

TargetScan software is dedicated to the analysis of mammalian miRNA target genes. Using this software, m7G-related mRNAs targeting miRNAs (m7G-related miRNAs) were predicted. The expression matrix of m7G-related miRNAs was extracted using R (version 3.4.4). The m7G-related miRNA expression matrix was normalized using R software "limma" package. The “edgR” package performed differential analysis of the normalized m7G-related miRNAs expression matrix between LUAD and normal tissues, and obtains m7G-related differentially expressed miRNAs (DEmiRs). Taking |logFC(fold change)|> 1 and adj. *p* < 0.05 as the screening criteria, the *p* value was adjusted using the Benjamini-Hochberg(BH) method. The correlation of m7G-related DEmiRs with patient overall survival (OS) was assessed through univariate Cox proportional hazards regression analysis using the survival coxph function of the R package. Lasso-penalized Cox regression was used to build an m7G-related prognostic miRNA signature. The risk score was calculated as follows: Risk score = e^sum(each miRNA’s expression * corresponding coefficient)^. Based on the median risk score, the patients were divided into high- and low-risk groups. The R software of the Rtsne package was used to perform principal component analysis (PCA) and t-SNE analysis of m7G-related DEmiRs based on the constructed model. A time-dependent receiver operator characteristic (ROC) curve analysis (1-, 3-, and 5-year) was conducted to assess the prediction accuracy of the signature using the survival and time ROC R package. The area under the curve (AUC) was used to measure the survival predicting the efficacy of the prognostic model. Calibration curve is an important indicator for evaluating models. Through the Hosmer–Lemeshow test, the *p* value was divided into 3 equal parts, and the difference between the predicted value and the actual value of each equal fraction was found. The above steps were completed through the “pec” package of the R software. The Nomogram transformed complex regression equations into visual graphs, making the results of predictive models more readable and convenient for patient evaluation. We used the Bootstrap self-sampling method and the modeling data to verify the predictive effect. This analysis was performed through the R languages “survival” and “rms” again.

The infiltration levels of 16 types of immune cells were quantified by single-sample gene set enrichment analysis (ssGSEA) using the gsva package in R software. At the same time, the ESTIMATE algorithm was used to evaluate the tumor microenvironment, including immune, stromal, and ESTIMATE scores. Spearman’s correlation method was used to evaluate the correlation between the risk score and the other variables. Pearson’s correlation was applied to detect correlations between variables of interest. Statistical significance was set at *p* < 0.05. The R software packages ggpubr and ggcorrplot were used to perform correlation analysis.

### Predictive chemosensitivity drug analysis

The United States National Cancer Institute (NCI) offers a panel of 60 human tumor cell lines (NCI-60 panel, https://dtp.cancer.gov/discovery_development/nci-60/) [10]. The R software package, pRRophetic, was used to predict METTL1 and WDR4 chemosensitivity based on a public pharmacogenomics database, Genomics of Drug Sensitivity in Cancer. The drug responses for 263 FDA-approved or clinical trial drugs were used in the correlation analysis.

### Statistical analysis

M7G-related DEmiRs were compared between LUAD and normal tissues using Wilcoxon test. The two-sided chi square test for independent proportions will be used for proportion comparisons. Mann–Whitney test was used to compare ssGSEA scores of high-risk and low-risk immune cells, and *p*-value was adjusted using the BH method. The Kaplan–Meier method was used to evaluate the significant difference in OS between the high- and low-risk groups. Univariate and multivariate Cox regression analyses were used to identify independent risk factors for LUAD. Spearman’s correlation method was used to evaluate the correlation between the risk score and the other variables. Pearson or Spearman correlation analysis was applied to detect correlations between variables of interest. Statistical significance was set at *p* < 0.05.

## Results

**Fig. 1:**
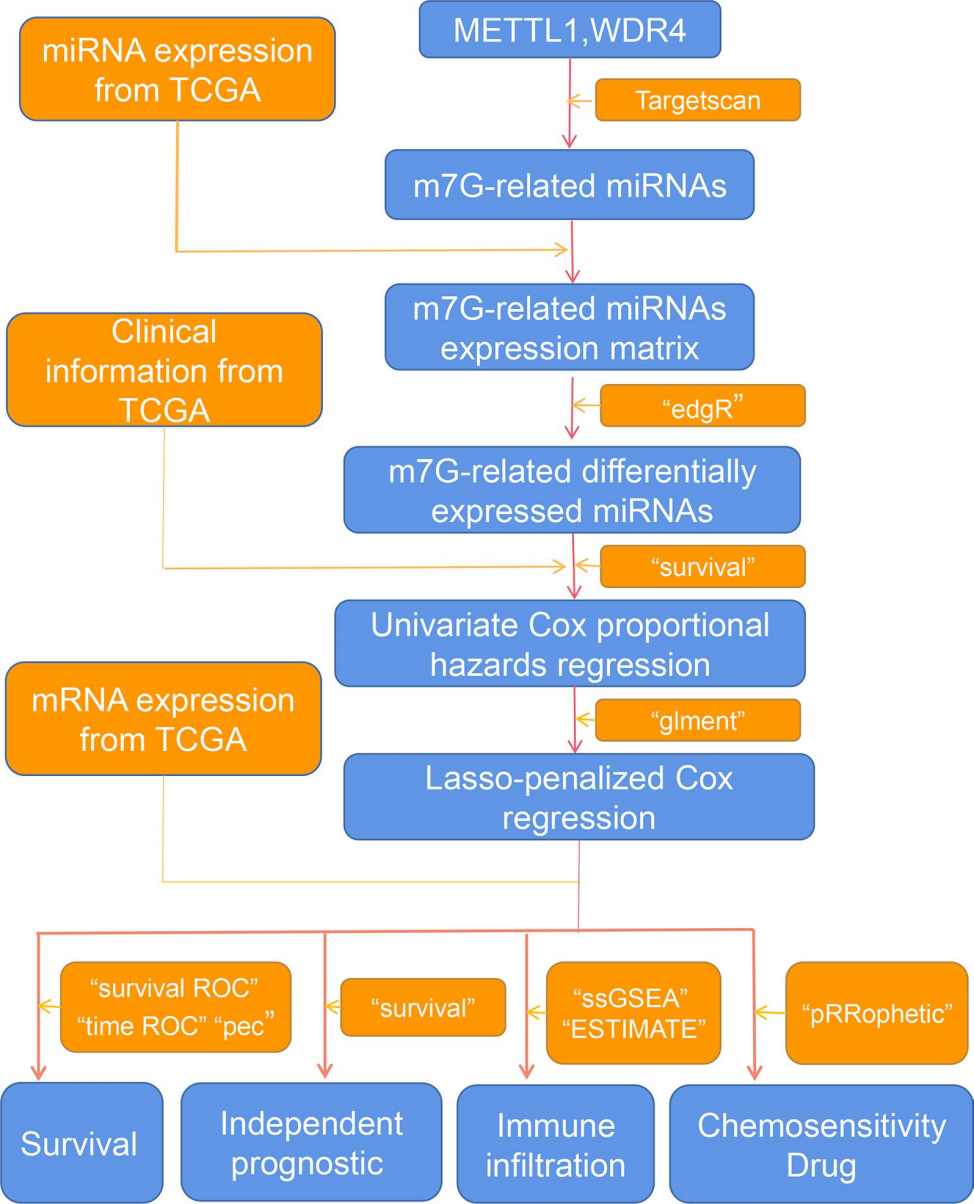
Technical roadmap of the study

The flow diagram of the study is shown in Fig. 1. The raw data consisted of mRNA (497 cases of LUAD and 54 cases of normal lung tissue) and miRNA(521 cases of LUAD and 46 cases of normal lung tissue) sequencing information of LUAD from The Cancer Genome Atlas (TCGA) database.

### Identification of prognostic m7G-related differential miRNAs

**Fig. 2:**
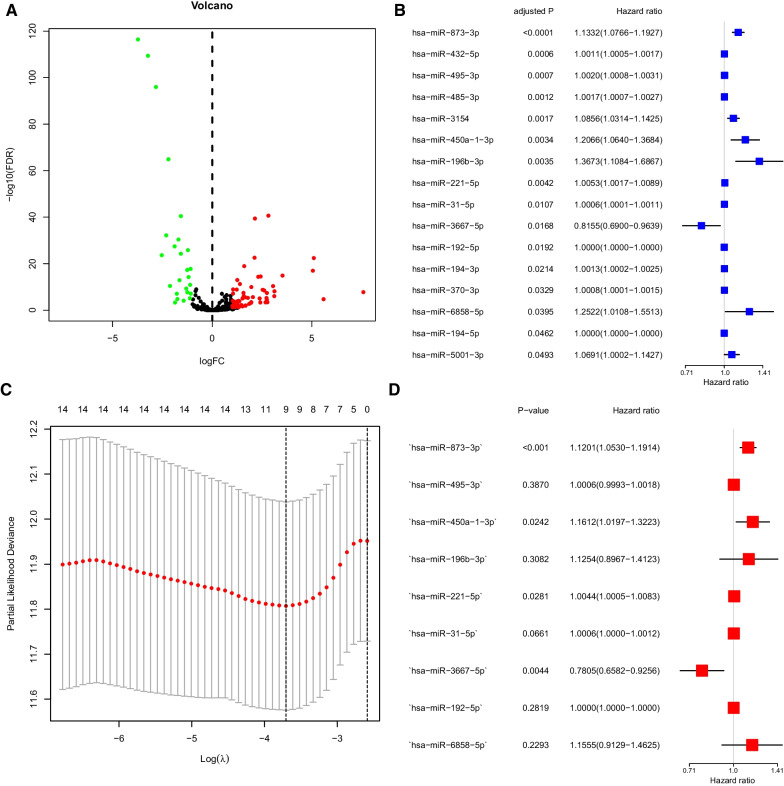
Model establishment. **A** Differential analysis, volcano plot represented the 88 m7G-related differentially expressed miRNAs between normal and lung adenocarcinoma (LUAD) tissues, of which the red was upregulated and the green was downregulated; **B** Univariate Cox proportional hazards regression analysis of overall survival of the correlation of m7G-related DEmiRs; **C** Partial likelihood deviance of overall survival for the LASSO coefficient profiles.; **D** Multivariate Cox proportional hazards regression analysis of overall survival of the correlation of m7G-related DEmiRs

Using TargetScan software, 792 miRNAs (m7G-related miRNAs) targeted by METTL1 and WDR4 were selected (Additional file 3: Table S1). Differential analysis revealed 88 m7G-related DEmiRs (63 upregulated and 25 downregulated) in normal and LUAD tissues (Fig. 2A). In univariate survival analysis, 16 m7G-related DEmiRs were associated with OS (Fig. 2B).

### Establishment of a prognostic model

**Fig. 3:**
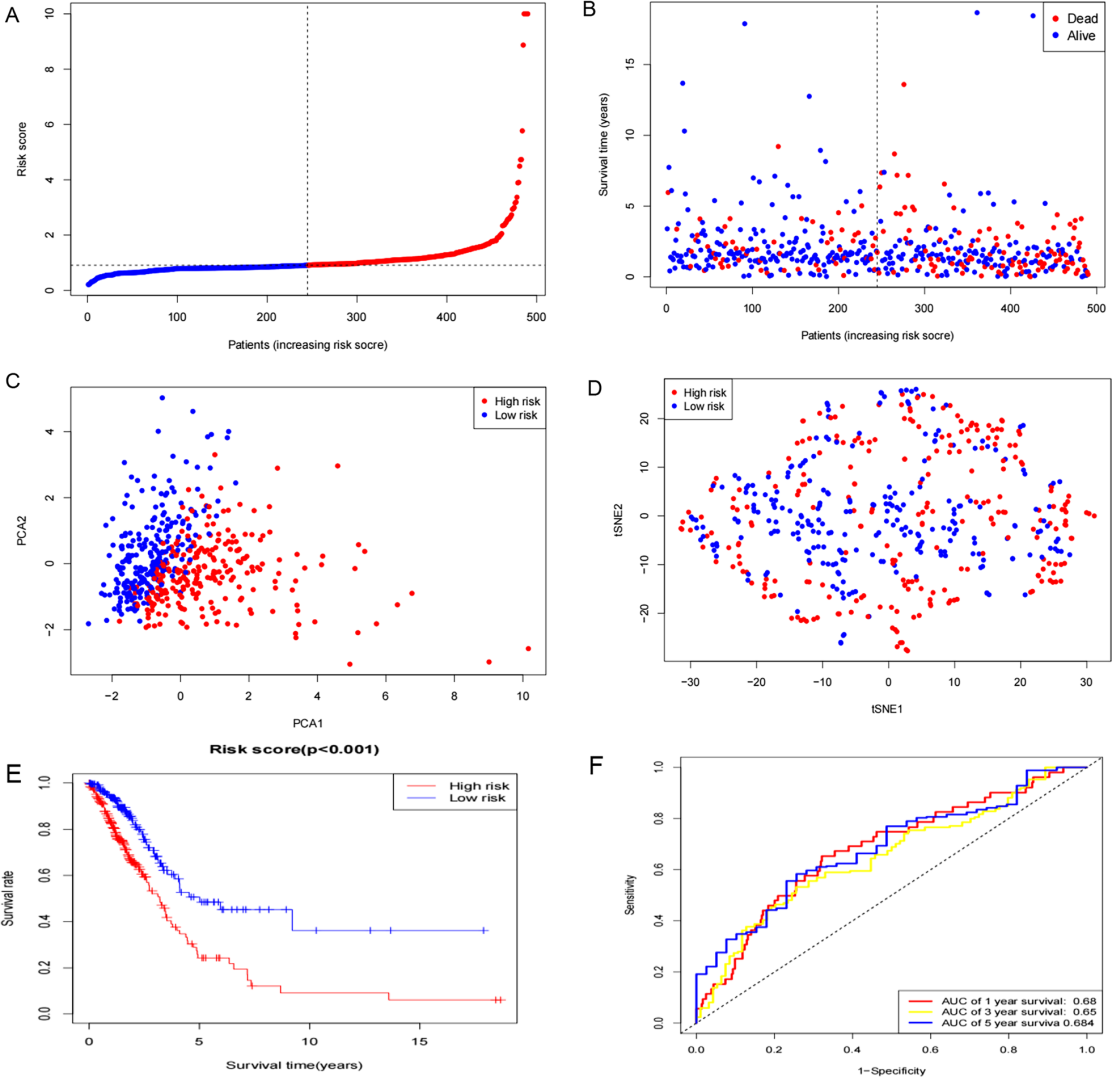
Prognostic analysis of the 9-miRNAS. **A** The distribution and median values of the risk scores in different groups.; **B** Distribution of risk scores for the high- and low-risk groups; **C** Principal component analysis separated samples between high- and low-risk; **D** t-SNE analysis of samples between high- and low-risk; **E** Kaplan–Meier curves for overall survival of patients in the high- and low-score groups; **F** The area under time-dependent receiver operator characteristic curves for overall survival

LASSO-Cox regression was performed to select the most effective variables from the 16 m7G-related DEmiRs to construct multivariate prediction models (Fig. 2C). When ʎ = 0.0246, nine m7G-related DEmiRs were selected (Fig. 2D). The prediction model signature was constructed as follows: Risk score = (0.095 × expression level of miRNA-3191-5p) + (4.001296e−04 × expression level of hsa-miR-495-3p) + (0.107 × expression level of hsa-miR-450a-1-3p) + (0.085 × expression level of hsa-miR-196b-3p) + (3.171833e−03 × expression level of hsa-miR-221-5p) + (4.475949e−04 × expression level ofhsa-miR-31-5p) + (− 0.156 × expression level of hsa-miR-3667-5p) +  6.897294e −07 × expression level of hsa-miR-192-5p) + (0.044 × expression level of hsa-miR-6858-5p). All patients were divided into two groups based on the median risk score: high-risk (n = 245) and low-risk (n = 245) (Fig. 3A, B). As expected, higher survival rates were observed in the low risk group than in the high-risk group. The results of PCA and t-SNE analysis showed that the high- and low-risk groups were well-distributed in two different directions (Fig. 3C, D). In survival analysis based on the risk score, the low-risk group showed better survival (*p* < 0.001, Fig. 3E). The area under the ROC receiver operating characteristic curve indicates that the performance of signature in 1-,3-, and 5-year survival predictions were 0.68, 0.65, and 0.683, respectively (Fig. 3F).

### Independent prognostic value of the signature

**Fig. 4:**
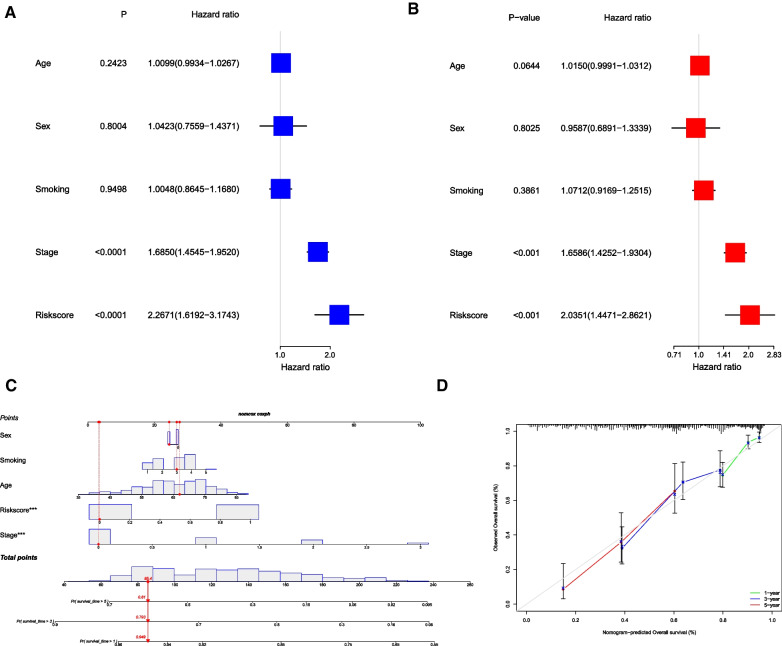
Signature validation. **A** Univariate Cox proportional hazards regression analysis of overall survival of the correlation of clinical characteristics and risk score; **B** Multivariate Cox proportional hazards regression analysis of overall survival of the correlation of clinical characteristics and risk score **C** Overall survival nomogram for LUAD patients; **D** Calibration plots for the nomogram

To determine whether the risk score is an independent prognostic factor for LUAD, univariate and multivariate Cox regression analyses were performed on the clinical characteristics and risk score. Univariate Cox regression analysis showed that the risk score was significantly associated with OS (*p* < 0.001, HR = 2.267, 95% CI 1.619–3.174, Fig. 4A). Multivariate regression analysis showed that risk score was an independent predictor of OS (*p* < 0.001, HR = 2.035, 95% CI 1.447–2.862, Fig. 4B). We generated a nomogram plot of the clinicopathological features and risk scores (Fig. 4C). The nomogram plot suggested that patients with lower risk scores and younger and lower stages had better survival rates. The calibration curves at 1, 3, and 5-year also illustrate the high predictive power of the structure (Fig. 4D).

**Fig. 5:**
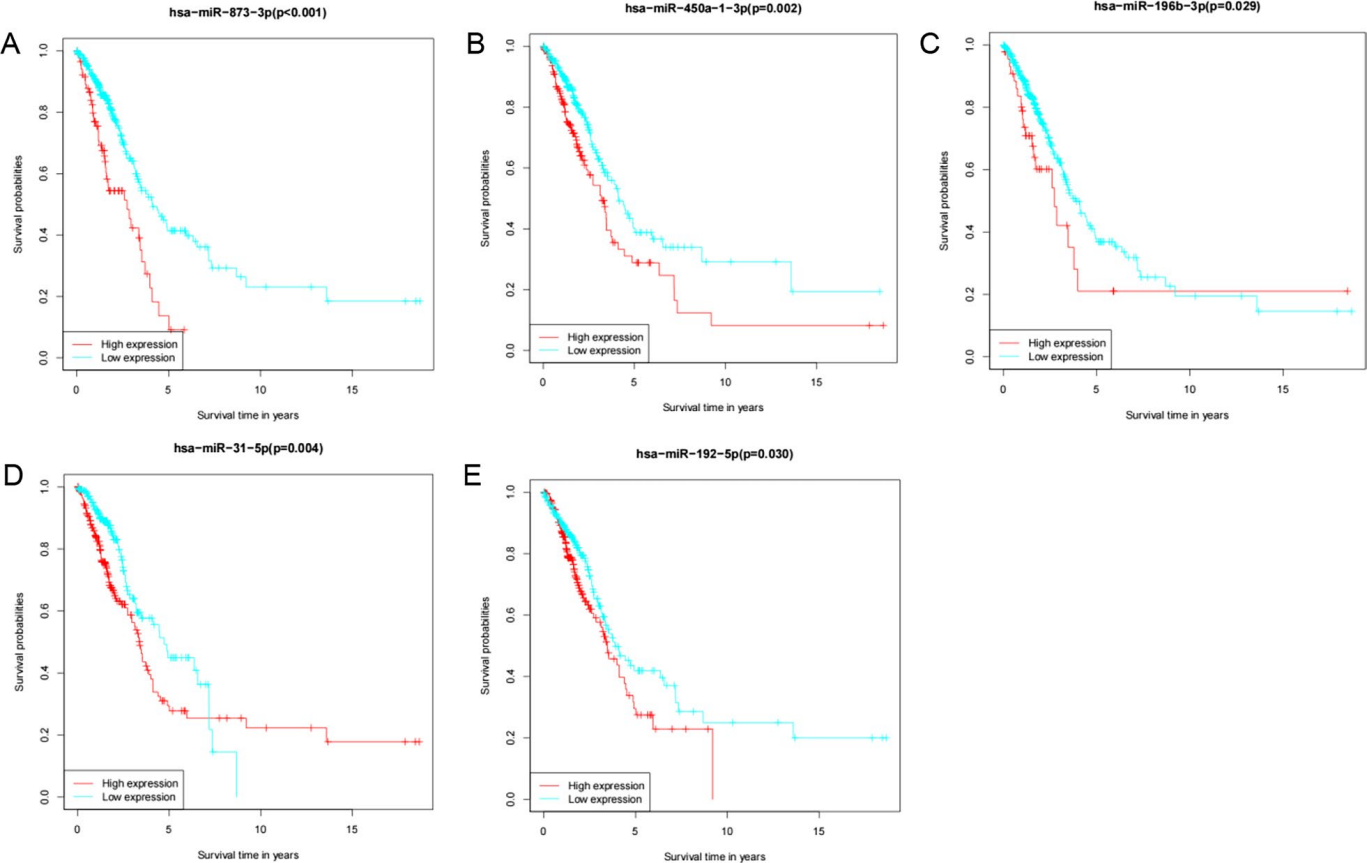
Kaplan–Meier survival analysis of miRNAs for the overall survival in lung adenocarcinoma (LUAD) patients, red reprented high expression, and green represented low expression. **A** hsa-miR-873-3p; **B** hsa-miR-450a-1-3p; **C** hsa-miR-196b-3p; **D** hsa-miR-31-5p; **E** hsa-miR-192-5p

Survival analysis of the nine m7G-related DEmiRs used to construct the prognostic model revealed that hsa-miR-873-3p (*p* < 0.001), hsa-miR-450a-1-3p (*p* = 0.002), hsa-miR-196b-3p (*p* = 0.029), hsa-miR-31-5p (*p* = 0.004), and hsa-miR-192-5p (*p* = 0.030) had significant prognostic significance for LUAD, and all showed better survival times at low expression (Fig. 5A–E). Moreover, these nine m7G-related DEmiRs were significantly highly expressed in LUAD (*p* < 0.05, Additional file 1: Fig. S1).

The risk score was only correlated with tumor stage in terms of clinical characteristics. The risk score was higher in the high stage (stages III-IV) than in the low stage (stages I–II) (*p* = 0.0031, Additional file 2: Fig. S2).

### Immune correlates analysis

**Fig. 6:**
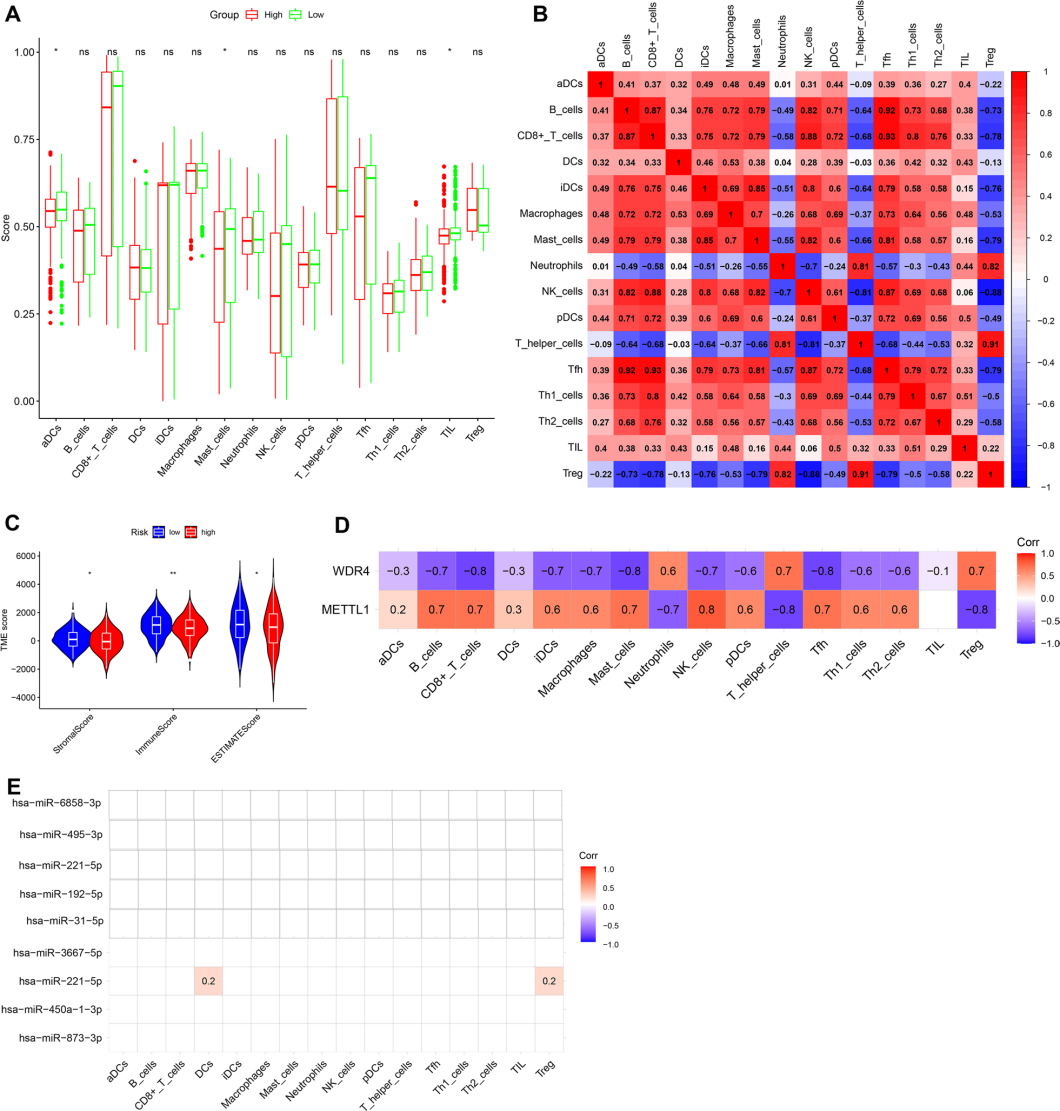
Immune cell infiltration. **A** Immune status of immune cells between high- and risk-score; **B** Correlation analysis of immune cells; **C** Immune status of tumor microenvironment between high- and risk-score; **D** Correlation analysis of METTL1 and WDR4 exprerssion wirh immune cells; **E** Correlation analysis of m7G related miRNA exprerssion wirh immune cells

Using the ssGSEA method, the infiltration of 16 immune cells was analyzed using the ssGSEA score as the standard. Between the high- and low-risk groups, activated dendritic cells (aDCs), mast cells, and tumor-infiltrating lymphocytes (TIL) showed significant differences (adj. *p* < 0.05) (Fig. 6A). The correlation analysis results of 16 immune cells showed that Treg and T helper cells showed a strong positive correlation (R = 0.91). Treg and NK cells showed a strong negative correlation (R = − 0.88) (Fig. 6B). The results from the ESTIMATE algorithm showed that the immune score, stromal score, and ESTIMATE of the tumor microenvironment were statistically different between the high- and low-risk groups (Fig. 6C). With the exception of TIL, METTL1 and WDR4 showed a certain degree of correlation with the remaining immune infiltrating cells. WDR4 showed a strong negative correlation with CD8^+^ T cells, mast cells, and T follicular helper (Tfh) (R = − 0.8, *p* < 0.05). METTL1 showed a strong positive correlation with natural killer (NK) cells (R = 0.8, *p* < 0.05) and a strong negative correlation with T helper cells and regulatory T [Treg] cells (R = − 0.8, *p* < 0.05) (Fig. 6D). Only hsa-miR-221-5p was positively correlated with Dendritic cells (DCs) and Treg Among the miRNAs used to construct the prognostic model (R = 0.2, *p* < 0.05; Fig. 6E).

### METTL1 and WDR4 expression and cancer cell sensitivity to chemotherapy

**Fig. 7:**
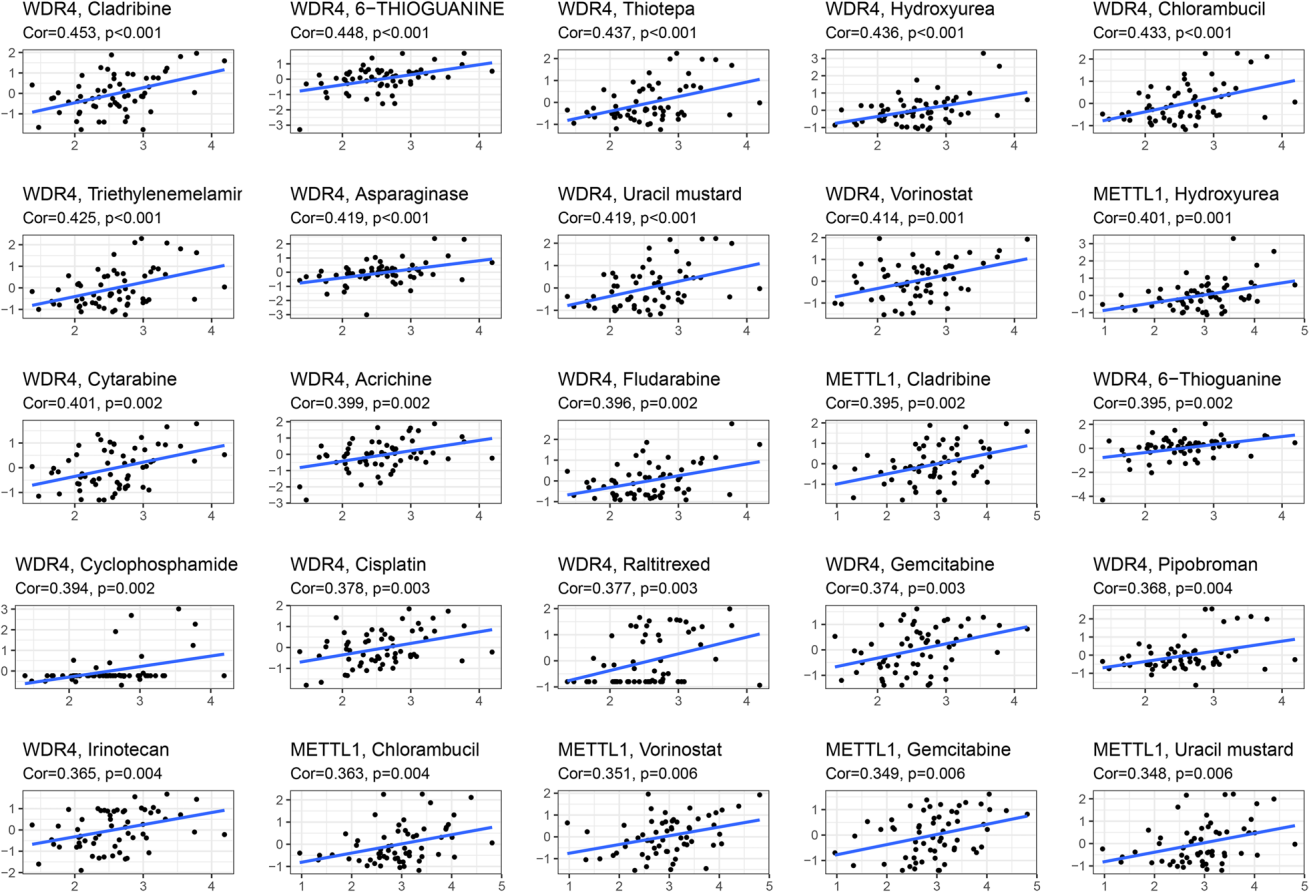
Scatter plot of expression of METTL1 and WDR4 with chemosensitivity

We used the NCI-60 cell line to investigate the sensitivity of METTL1 and WDR4 to chemotherapy. The results showed that the expression of these two genes was positively correlated with chemotherapy drugs (*p* < 0.01, Fig. 7). For example, increasing the expression of WDR4 significantly improved the sensitivity to cladribine, 6 − THIOGUANINE, Thiotepa, hydroxyurea, and other drugs. We also found that all drugs that responded to METTL1 responded to WDR4 and were positively correlated.

## Discussion

Lung cancer is a malignant tumor of the bronchial epithelium, and its morbidity and mortality are extremely high worldwide [11]. Owing to the high morbidity and mortality of lung cancer, the discipline of integrating and analyzing the data of lung cancer patients through public databases and bioinformatics methods has gradually emerged in recent years. In the past few decades, the application of high-throughput technology in molecular biology has not only changed the research methods of biology and biomedicine but also laid the foundation for the development of bioinformatics. miRNAs are involved in various biological processes of tumor evolution, including tumor cell proliferation, apoptosis, cell cycle regulation, adhesion, and angiogenesis induction [12]. Therefore, the abnormal expression of miRNAs is closely related to the occurrence and development of tumors. When its expression is upregulated or downregulated, it can be used as a tumor-promoting or tumor-suppressing factor to target and regulate the gene expression of downstream signaling pathways [13], and plays an important role in various malignant tumors, including lung cancer [14].

Although some studies have shown the regulatory role of m7G methylation in the occurrence and development of lung cancer [14], there are no reports on the use of m7G-related miRNAs to construct a prognostic model. We found 88 m7G-related DEmiRs (11.1%) among 792 m7G-related miRNAs between LUAD and normal tissues. Univariate regression analysis revealed that the 16 m7G-related DEmiRs were associated with OS. LASSO regression was further selected, and nine m7G-related DEmiR (hsa-miR-873-3p, hsa-miR-495-3p, hsa-miR-450a-1-3p, hsa-miR-196b-3p, hsa-miR-221 -5p, hsa-miR-31-5p, hsa-miR-3667-5p, hsa-miR-192-5p, and hsa-miR-6858-5p) prognostic models were constructed. The m7G-related miRNA signature predicted the AUC of 1-, 3-, and 5-year survival of LUAD to be 0.68, 0.65, and 0.684, respectively. Patients were divided into high- and low-risk groups based on the median risk score and the expression levels of the nine m7G-related DEmiRs. We found that the patients in the high-risk group had lower immune cell infiltration and shorter survival times. Moreover, the risk score can be used as an independent predictor of LUAD prognosis. The expression of the nine m7G-related miRNAs built into the model was higher in LUAD tissues than in normal tissues and showed statistical differences. In particular, the expression of hsa-miR-196b-3p in normal lung tissue was extremely low. The results of differential analysis of immune cell infiltration between the high- and low-risk groups showed that the level of immune cell infiltration in the high-risk group was generally lower than that in the low-risk group. Immune cells closely related to LUAD showed statistically significant differences (DCs and Tregs).

The M7G-related miRNAs that constitute the prognostic model can be roughly divided into three categories. The first type has a promoting effect on tumors (hsa-miR-196b-3p, hsa-miR-221-5p, and hsa-miR-31-5p). MiR-196b-3p, as the degraded product from the 3 prime of its precursor miR-196b, significantly increased expression in recurrent epithelial ovarian cancer [15]. JEONG et al. [16] found that hsa-miR-196b-3p promotes the progression of prostate cancer. MiR-196b enhanced NSCLC cell migration and invasion by downregulating GATA6 [17]. A study by Liao et al*.* [18] showed that hsa-miR-221-5p is highly expressed in NSCLC and that this high expression promotes the malignant phenotype of NSCLC cells, which is consistent with our findings. Hsa-miR-31-5p is widely accepted as an oncogenic miRNA that is involved in the proliferation, migration, invasion, and chemosensitivity of various tumor cancer cells [19–22]. Hsa-miR-31-5p is ectopically upregulated in various malignancies [23]. Studies have shown that miR-31-5p expression is associated with the Warburg effect in lung tumorigenesis [24, 25]. Yu F found that hsa-miR-31-5p significantly accelerated the metastasis of LUAD cells in vitro and in vivo [26]. In this study, it was found that hsa-miR-31-5p was significantly upregulated in HExo and bound to protein 2 by targeting a specific AT-Rich sequence (SATB2), and activation of the MEK/ERK pathway promotes tumor progression [26]. The second type has an inhibitory effect on the proliferation and invasion of tumor cells (hsa-miR-873-3p, hsa-miR-495-3p, hsa-miR-192-5p, and hsa-miR-3667-5p). Some studies have proposed that hsa-miR-873-3p mediates NFATC2 and GSK3B (Wnt pathway) to regulate LUAD cell proliferation and invasion [27]. Wnt signaling is an oncogenic driver in lung, breast, liver, and ovarian cancers [28]. In NSCLC, hsa-miR-495 binds to the HOXC93-UTR region and inhibits its expression, suggesting that it plays a tumor suppressor role in the MIR-495/HOXC9 pathway [29]. In our study, the low-expression hsa-miR-495-3p group had better survival time and was highly expressed in LUAD, suggesting that it is a negative regulator. Hsa-miR-192-5p is involved in a variety of human diseases, especially various cancers, including lung, liver, cervical, and breast cancers [30, 31]. However, they play different roles in different tumors. Similar to HCC, prostate cancer [32] promotes tumor erosion. Hsa-miR-192-5p inhibits NSCLC cell proliferation through the Wnt/β-catenin signaling pathway [33]. Few studies have focused on the functions of hsa-miR-3677 in human cells. Studies have shown that hsa-miR-3677 is inversely associated with overall survival in HCC patients [34]. Targeting SphK1 via hsa-miR-3677 has also been shown to inhibit the progression of human osteosarcoma cells [35]. However, there are few reports of related research on lung cancer.

The last category is almost unreported. These miRNAs were proposed for the first time to play different roles in LUAD cells (hsa-miR-450a-1-3p and hsa-miR-6858-5p). Hsa-miR-450a-1-3p is widely expressed in various tissues, but little is known about its biological functions. Only studies have shown that its expression is different in endometrial carcinosarcoma and prostate cancer [36]. Some studies have found that the target gene regulated by hsa-miR-450a-1-3p is Bub1 [37]. Spindle assembly checkpoint (SAC) lesions caused by Bub1 dysregulation play an important role in tumorigenesis [37]. Hsa-miR-6858-5p is a key regulator of melatonin (MEL) suppresses the malignant properties of GBM [38]. Hsa-miR-450a-1-3p, hsa-miR-6858-5p regulate METTL1 of m7G-related genes in lung cancer. METTL1 can function in LUAD cells through the AKT/mTORC1 pathway, and upregulation of METTL1 occurs early in LUAD development [39]. We speculated that hsa-miR-450a-1-3p and hsa-miR-6858-5p may also act in the early stages of LUAD through the AKT/mTORC1 pathway. However,the specific functions need to be studied further. However, our study found that hsa-miR-450a-1-3p was highly expressed in LUAD, whereas low expression was associated with better survival time. Hsa-miR-450a-1-3p positively regulates the proliferation of LUAD cells.

The risk scores were more strongly associated with a higher clinical stage. This result further suggests that higher risk scores and later clinical stages are associated with poorer survival rates. Immune-infiltrating aDCs, mast cells, and TIL showed higher immune scores and differences in the low-risk group. This could also explain the better survival of the patients in the low-risk group. Analysis of the tumor microenvironment and risk score also supported the conclusion that patients in the low-risk group had longer survival times. METTL1 was positively correlated with aDCs, mast cells, and TIL, while WDR4 was the opposite. These three types of immune cells were strongly and positively correlated with each other. This indicates that the immunoregulatory effect of METTL1 is greater than that of WDR4 in the tumor microenvironment. In a previous analysis, METTL1 was significantly elevated in the early stages of LUAD, promoting tumors. In some studies, it was found that the expression of METTL1 did not change significantly from LUAD stage I–stage IV and was in a state of high expression [40]. M7G methylation mediated by METTL1 promotes tumor proliferation in the early stages of LUAD [39]. However, with the development of tumors, the promotion effect is inhibited owing to the intervention of a certain mechanism, but the immune mechanism of METTL1 is stimulated. Dynamic modification of RNA enables cells to respond rapidly to changes in the external environment and adapt to the changing microenvironment. The METTL family can function through a variety of mechanisms to regulate the maturation, differentiation, and function of immune infiltrating cells, thereby affecting the occurrence of immune responses and stability of the immune system [41, 42]. METTL3 plays an important role in the normal differentiation of Tn cells, maintenance of Treg inhibitory function, activation of aDC, polarization of macrophages, and expression of inflammatory factors [42]. In the colorectum, METTL14 expression levels affect the infiltration of CD8 + T cells [43].

Using the NCI-60 database, we found that with the increased expression of METTL1 and WDR4, the sensitivity of some FDA-approved chemotherapeutics was also enhanced. Cisplatin and gemcitabine are commonly used as second-line drugs for the treatment of non-small cell lung cancer. However, drugs such as cladribine and thiotepa have also been used to treat other tumors. These drugs may provide new insights into the treatment of LUAD.

However, there are still some limitations in this paper. We provided the prognostic relationship of nine N7-Methylguanosine related miRNAs with LUAD patients only based on the analysis of public databases, and we only theoretically analyzed the effect of expressions of METTL1 and WDR4 on chemotherapeutic agents in LUAD patients. This study lacks external database and experiments in vivo or in vitro to validate the above findings. All of these things need to be improved later.

## Conclusion

Overall, our study established a novel prognostic signature consisting of 9 m7G-related miRNAs. Multidimensional analysis revealed that singularity was an independent prognostic factor in patients with LUAD. However, there are still theoretical and experimental deficiencies in the regulation of m7G methylation during the development of LUAD, which is worthy of further exploration.

## Supplementary Information

**Additional file 1. Supplementary Figure 1**. M7G related miRNA exprerssion between normal and tumor tissus.

**Additional file 2. Supplementary Figure 2**. The relationship between risk score and clinical features.

**Additional file 3. Supplementary Table 1**. 792 miRNAs targeted by METTL1 and WDR4.

## Data Availability

The datasets used and/or analysed during the current study available from the corresponding author on reasonable request.
