# Supplementary figures and images for: A noval prognostic signature of the N7-methylguanosine (m7G)-related miRNA in lung adenocarcinoma
Source: BMC Pulm Med. 2023 Jan 12;23:14. doi: 10.1186/s12890-022-02290-7 (PMC9838007; doi:10.1186/s12890-022-02290-7)

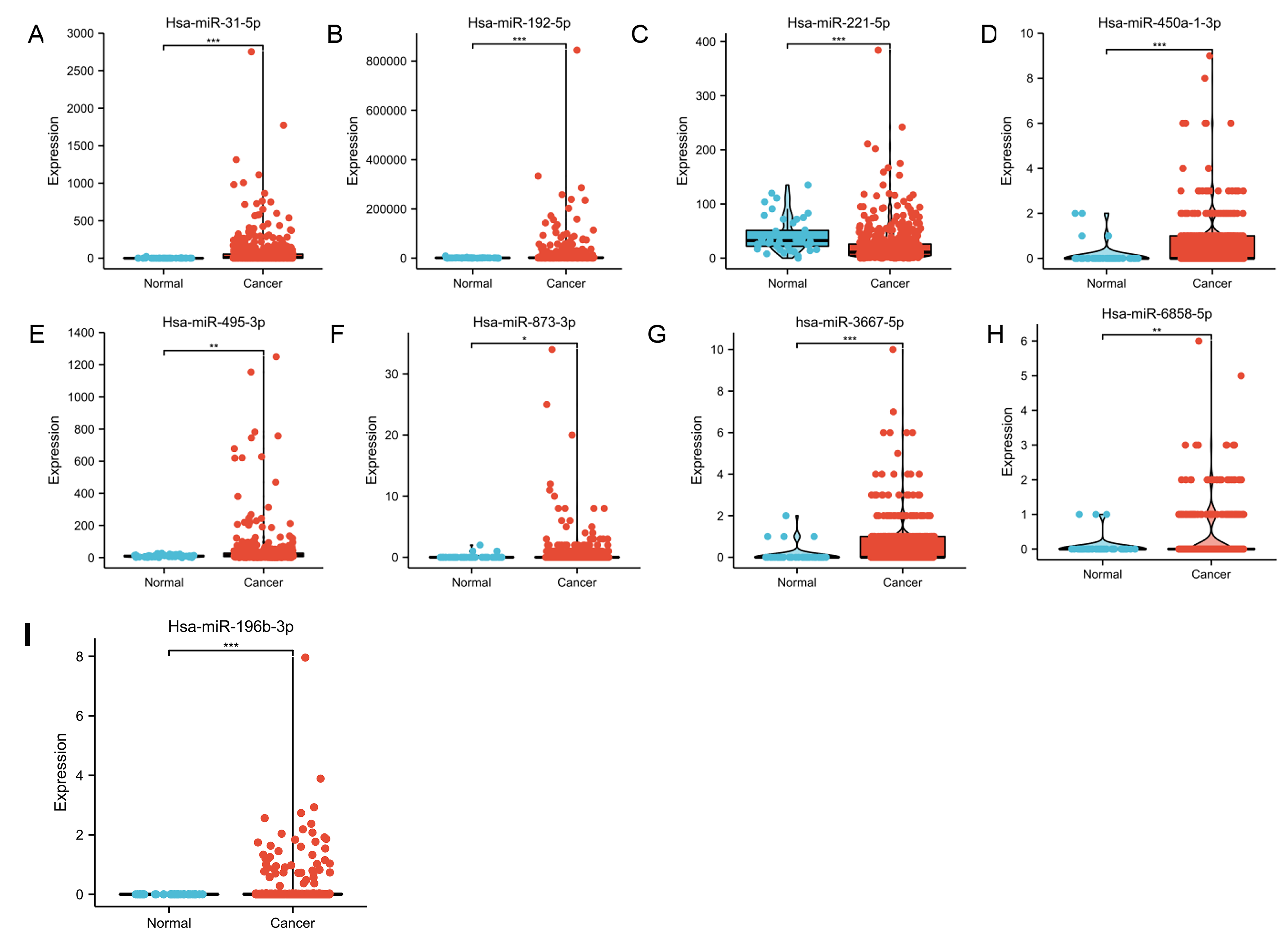

Supplement: Supplementary file 1 — Additional file 1. Supplementary Figure 1. M7G related miRNA exprerssion between normal and tumor tissus. [file 12890_2022_2290_MOESM1_ESM.tif]

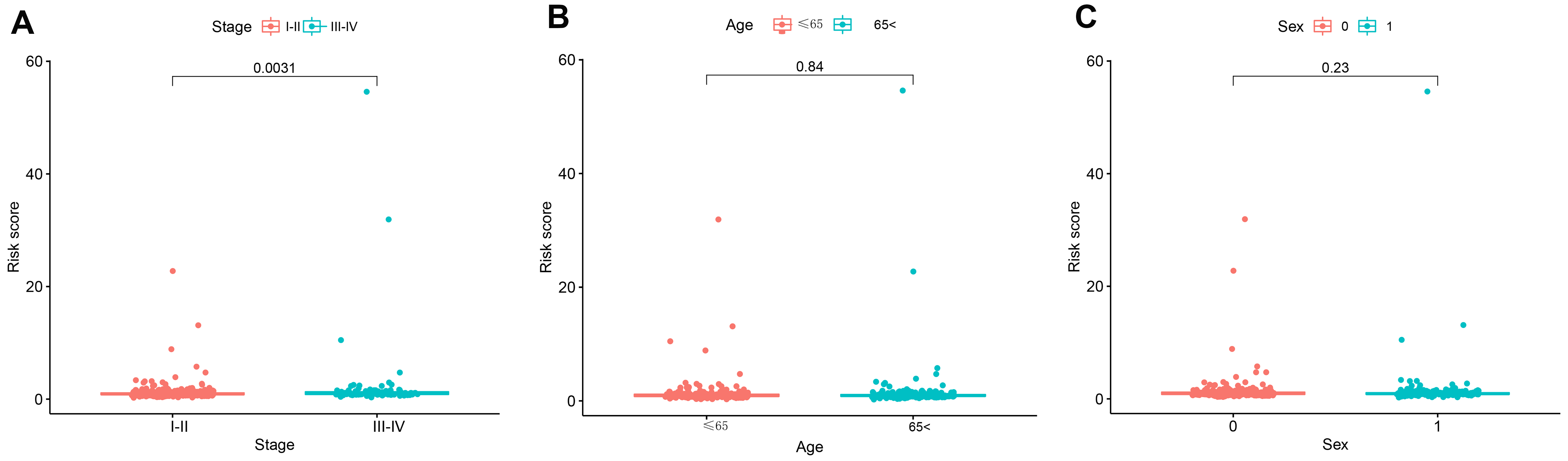

Supplement: Supplementary file 2 — Additional file 2. Supplementary Figure 2. The relationship between risk score and clinical features. [file 12890_2022_2290_MOESM2_ESM.tif]
